# Beyond the Tram Lines: Disability Discrimination, Reproductive Rights and Anachronistic Abortion Law

**DOI:** 10.1093/ojls/gqad025

**Published:** 2023-12-02

**Authors:** Sally Sheldon

**Keywords:** disability discrimination, abortion law, reproductive rights, Abortion Act 1967, anti-abortion campaigning, *Crowter v SSHSC*

## Abstract

This article takes as its starting point the recent case of *Crowter*, which challenged the law permitting provision of abortion on the grounds of fetal anomaly. It begins by briefly locating the case within a longer ‘biography’ of the Abortion Act 1967, casting important light on the issue raised within it. It then focuses in detail on the claims made in *Crowter*, exploring how important moral, social and political concerns with disability discrimination were refracted through an anti-abortion lens as they were translated into legal argument. As a result, the legal remedies sought were simultaneously disproportionate and insufficient to address the harms described. Whilst agreeing that the Abortion Act reflects anachronistic and discriminatory understandings of disability and is overdue reform, the article argues that a response that fully reflects modern ethical values will require more radical change than envisaged in *Crowter*, and that this must refuse an opposition between the rights of pregnant and disabled people.

If abortion is mentioned, up go the barricades to defend the right to life or the right to choose. On no other issue is there such a dialogue of the deaf in this Chamber, with the slightest concession to one side being seen as enabling the wholesale destruction of the other … The House is normally left defending the Abortion Act 1967—with all its weakness, which are acknowledged even by some of its major proponents—as though it were holy writ.^1^

## 1. Introduction

Given a blank sheet of paper, no modern lawyer, ethicist, theologian, campaigner or politician of any political hue would draft the current British abortion laws, which consist of a tangle of archaic, overlapping, punitive criminal prohibitions, only partly mitigated by a statute that is itself shaped by the very different moral norms and clinical realities of the late 1960s. While abortion is now extremely safe and very common, this very old statutory framework has nonetheless proved curiously resilient in the face of repeated proposals for its reform. Following the failure of his own reform bill in the late 1980s, Lord Alton complained that the Abortion Act 1967 appears to have become set in concrete as a ‘great untouchable’,^2^ with debate about it preceding along ‘tram lines that never converge’.^3^

In this article, I focus on a recent important legal challenge to one part of this legal framework: a call for reform of section 1(1)(d) of the Abortion Act 1967, which permits termination without time limit where there is a ‘substantial risk’ of a future child ‘suffering’ ‘serious handicap’ as an exception to a more general prohibition on abortion. The challenge was brought by Heidi Crowter, a young disability rights campaigner with Down’s syndrome,^4^ Aidan Lea-Wilson, a child with Down’s syndrome, and Aidan’s mother, Máire Lea-Wilson, who was offered the option of termination following the prenatal diagnosis of Aidan’s Down’s syndrome at 34 weeks.^5^ The claimants presented their challenge as necessary to end discrimination against disabled people and achieve ‘parity in the law’,^6^ with Crowter explaining:

At the moment in the UK, babies can be aborted right up to birth if they are considered to be ‘seriously handicapped’. They include me in that definition of being seriously handicapped—just because I have an extra chromosome! Can you believe that? What it says to me is that my life just isn’t as valuable as others, and I don’t think that’s right.^7^

I use *Crowter* to illustrate a broader claim: that the ‘tram lines’ of the current political debate have tended to entrench a reductive focus on attacking or defending the Abortion Act in a way that imagines piecemeal reform, ignoring and obscuring the need for more fundamental change. Drawing on extensive archival research, I locate the case within a longer history of contestation regarding the Abortion Act, noting the extent to which it has become an important focus for wider concerns regarding gender and familial norms, and the respective roles of science and religion in shaping our understanding of the world.^8^ Notably, anti-abortion and Christian campaigning groups have had a hand in almost all litigation concerning the Abortion Act in its 50-year history, reporting alleged abuses to police, maintaining a spotlight on decisions to investigate and prosecute, encouraging and supporting claimants in civil cases and sometimes intervening directly.^9^ While not always winning the formal legal remedy requested, campaigners have often been remarkably effective in provoking and shaping public debate, influencing successive governments against relaxation of regulatory controls and sometimes achieving a chilling effect on clinical practice.^10^

Campaigners’ work has also contributed to the framing of abortion law reform debates, and one impact of this has been to conceal what might otherwise appear as widely shared common ground. Specifically in this case, I argue that, seen from the ‘tram lines’, the important moral and political concerns with disability discrimination raised by the *Crowter* claimants were refracted through a wider anti-abortion lens in the process of being translated into legal arguments.^11^ Within this framing of the legal issues, I argue, disability rights were pitched against abortion rights in a reductive zero sum game, which obscured important potential common ground between the two.

My argument proceeds as follows. First, I briefly outline existing abortion law, with an emphasis on the assumptions that shaped section 1(1)(d), before noting three broad trends that have influenced its shifting implementation and interpretation since 1967.^12^ This brief ‘biography’ of the Abortion Act keeps sight of two features that are important to understanding any written norms: first, they are rooted in the past, enshrining historically contingent values and practices; and second, as linguistic structures, they can take effect only through acts of interpretation and are thus simultaneously constantly evolving in ways that are influenced by broader social trends.^13^ This approach both validates and problematises the important claim made in *Crowter*: that the ‘obvious vice of s.1(1)(d) is that it is in conflict with modern attitudes towards disabled persons’.^14^ The Abortion Act 1967 was indelibly marked by the prevailing social norms and clinical practices of a specific historical moment, including—importantly for the *Crowter* claimants—morally repugnant, discriminatory assumptions and eugenic beliefs regarding disabled people. However, meaning has been given to it over time by an evolving cast of actors rooted in dramatically changing social, demographic, professional and institutional contexts. This cast list includes women seeking abortion services, doctors and other health professionals, politicians, campaigners, academics, journalists, judges and lawyers, whose own biographies have both shaped and been shaped by these engagements.^15^ Most notably for current purposes, the interpretation and implementation of the Abortion Act has evolved with an increasing emphasis on the responsibility of doctors to offer non-directive, supportive care to their patients; to facilitate their informed decision making and respect their choices; and to avoid discriminatory assumptions about what it means to live with a disability. Recognising this tension between the text of ageing legislation and its evolving interpretation and implementation is key to understanding the issues raised in *Crowter*.

Having first sketched this context, I move on to consider the arguments in *Crowter*. I locate their framing within the ‘tram lines’ of current, deeply polarised abortion debates and the legal reforms envisaged within a wider strategy of ‘chipping away’ at the current, ageing law to achieve its piecemeal reform. With the important moral, political and social concerns raised by the *Crowter* claimants refracted through an anti-abortion lens as they are translated into legal arguments, I argue that the kinds of legal reform discussed in the action are simultaneously disproportionate and insufficient to address the important moral and political harms they described, and unnecessarily framed in opposition to reproductive rights. I suggest that a legal and policy response that fully reflects modern ethical values of equality, diversity and inclusion will require more radical reform than envisaged in *Crowter*, and that it must refuse an opposition between the rights of pregnant and disabled people in favour of treating them as mutually reinforcing concepts.

## 2. Ageing Abortion Law and Changing Implementation

### A. Ageing Abortion Law

Access to abortion is governed by the oldest statutory framework governing any modern medical procedure.^16^ In England and Wales, the offence of ‘unlawful procurement of miscarriage’ is punishable by up to life imprisonment under section 58 of the Offences Against the Person 1861, a mid-Victorian statute characterised by overlapping offences, inconsistent sentencing provisions, harsh punishments and archaic language.^17^ The Abortion Act did not repeal this offence but carved out exceptions to it, rendering abortion lawful only under conditions of strict medical control:

(1) Subject to the provisions of this section, a person shall not be guilty of an offence under the law relating to abortion when a pregnancy is terminated by a registered medical practitioner if two registered medical practitioners are of the opinion, formed in good faith—
  (a) that the pregnancy has not exceeded its twenty-fourth week and that the continuance of the pregnancy would involve risk, greater than if the pregnancy were terminated, or of injury to the physical or mental health of the pregnant woman or any existing children of her family; or
  (b) that the termination is necessary to prevent grave permanent injury to the physical or mental health of the pregnant woman; or
  (c) that the continuance of the pregnancy would involve risk to the life of the pregnant woman, greater than if the pregnancy were terminated; or
  (d) that there is a substantial risk that if the child were born it would suffer from such physical or mental abnormalities as to be seriously handicapped.^18^

The history of the Abortion Act has been explored in significant detail elsewhere, so here I recall just a few aspects of direct relevance to *Crowter*.^19^ In the late 1960s, a recent German measles epidemic and the thalidomide scandal had shaped public opinion.^20^ A risk of fetal anomaly was widely seen as offering a respectable reason for termination, with public opinion favouring it where a child might be born ‘seriously deformed’,^21^ reflecting widely shared assumptions that the birth of a disabled child was a tragedy for all concerned, with ‘a lonely, outcast, and dependent future’ written on the child’s body.^22^ Abortion was seen as justified by the strain on parents whose lives may be ‘blighted by having to rear a grossly defective child’, and ‘perhaps secondly by consideration for the public purse’.^23^ It was also often deemed in the best interests of ‘mentally defective children’ themselves,^24^ reflecting the harsh realities of a time when disabled children were frequently housed in overcrowded, underfunded and geographically isolated homes treated as ‘dumping grounds for social undesirables’.^25^ Indeed, the perceived desirability of termination in such circumstances did much to legitimise abortion more generally, breaking a perceived association between unwanted pregnancy and illicit, non-marital sex, and framing abortion as a procedure that might be sought by respectable, married, middle-class women.^26^

While the provisions of the Abortion Act were extensively and fiercely debated, opposition to the fetal anomaly ground was relatively muted and focused largely on practical concerns rather than principled objections. Critics emphasised the limitations of prenatal testing: given a high number of false positive diagnoses and a 3–5% risk of provoking miscarriage, expanding prenatal testing risked ‘the slaughter of thousands of potentially healthy children to avoid the birth of a few deformed ones’.^27^ Understandings that challenged a dominant narrative of disability as a tragedy for all concerned were glimpsed only infrequently, through anecdotal accounts of the triumph of love over adversity in particular families, or stories of individuals who had heroically overcome impairments to lead successful lives. Still more rarely were the voices of disabled people themselves heard.^28^

If the views of disabled people were largely missing from political debates regarding the proposed abortion law, those of women were also badly underrepresented. In 1967, an emerging women’s liberation movement had not yet begun to call for abortion rights, there were only 26 female MPs and abortion was not generally understood as an issue on which women had any particular authority to speak.^29^ The need for abortion law reform was instead presented as a humanitarian measure to end the ‘tyranny of excessive fertility’ blighting the lives of working-class mothers^30^ and the stark social inequality that saw safe abortion as the prerogative of the rich.^31^ The Abortion Act was needed to bring abortion ‘into the hands of the medical profession’,^32^ protecting the beneficent exercise of clinical discretion, offering relief in limited deserving cases and ensuring the hygienic conditions that might eliminate unsafe, illegal abortion and consequential mortality and morbidity. It was argued that this would promote motherhood and family life,^33^ with abortion for fetal anomaly also serving this broader purpose, for—as one doctor informed the House of Commons—the woman who is forced to give birth to a disabled child will seldom allow herself to become pregnant again.^34^ The text of the Abortion Act was indelibly marked by these concerns.

Over the following five decades, debate would become starkly polarised, with Hansard recording over 60 occasions on which further attempts were made to reform abortion laws.^35^ This sustained contestation reflects, in part, the Abortion Act’s position on the shifting tectonic plates of a society undergoing a demographic revolution. The Act has been both artefact and driver of rapidly changing familial and gender norms; significant changes in the medical relationship, clinical practice and abortion technologies; and, importantly, an increasing rejection of religious norms in favour of an emphasis on science in ordering understandings of the world.^36^ It has served as a cipher for these broader concerns, with a significant impact on advocacy regarding it: with individual exceptions, those leading anti-abortion campaigns are now overwhelmingly likely to be people of Christian faith with fundamental moral objections to abortion.^37^ However, to optimise support in a society that has rapidly grown markedly more socially liberal and secular, they have tended increasingly to offer a grudging defence of the Abortion Act; to propose narrowly focused proposals for reform that seek to chip away at abortion access, whilst leaving the restrictions that it imposes otherwise intact; and to justify the need for such measures in terms of modernisation, the empowerment and protection of women, and civil liberties and human rights.^38^

Notwithstanding these frequent attempts at reform, the Abortion Act has survived largely intact, reflecting the reluctance of successive governments to give parliamentary time to an issue known to be divisive. Substantive amendments have been made to its text on only two occasions, including in 1990, when the upper time limit on abortions authorised under section 1(1)(d) was removed.^39^ This is the provision contested in *Crowter*.

### B. Changing Interpretation and Implementation

While the Abortion Act’s text has endured largely unaltered, its interpretation and implementation have been subject to profound change. First, abortion has been increasingly normalised as part of routine UK healthcare. Technological innovations have made it a far safer, technically easier procedure, performed ever earlier in pregnancy;^40^ telemedical services now mean that many women can manage their own abortions at home; almost all reported abortions are funded by the NHS;^41^ and, as modern Britons have gradually looked less to religion for guidance on moral issues, socially liberal attitudes towards abortion have become dominant. A large majority of British adults now believe that the law ‘should allow an abortion when the woman decides on her own she does not wish to have the child’;^42^ and up to one in three British women will terminate a pregnancy at some point in their lives.^43^

These changes have shaped ongoing political debates regarding abortion, in a way that is exemplified by changes within the parliamentary debates. Initially, opposition to the Abortion Act and proposals for wide-ranging restrictive reforms were led by politicians who stood on a centre ground of mainstream family-values Conservativism built on the bedrock of an unspoken Christian heritage.^44^ More recently, the cause has been championed by members of the Tory Cornerstone Group (including Nadine Dorries, Fiona Bruce, Jacob Rees-Mogg, Laurence Robertson and the late David Amess), which likewise emphasises ‘the spiritual values which have informed British institutions, her culture and her nation’s sense of identity for centuries, underpinned by the belief in a strong nation state’.^45^ However, these MPs are today distinguished from the parliamentary mainstream in the emphasis that they place on religious faith in driving their parliamentary work. Aware of working within an increasingly secular Parliament, they have tended to propose reforms narrowly focused on issues likely to optimise political support, and to justify them in a secular rhetoric of modernisation, civil liberties and the protection and empowerment of women.^46^

Second, with safe, legal abortion now available in cases of ‘substantial risk’ of ‘serious handicap’, dramatic technological advances followed in prenatal screening and testing. The goal of reducing the number of children born with Down’s syndrome was an important driver, underpinning a ‘silent revolution’ in antenatal care.^47^ The development of ultrasound greatly improved prenatal diagnosis of a growing range of anomalies, and its use to guide amniocentesis has significantly reduced the associated risk of miscarriage.^48^ Chorionic villus sampling, then maternal serum testing and, most recently, non-invasive prenatal testing (NIPT) allowed the offer of amniocentesis to be more accurately targeted, greatly reducing the number of women exposed to its associated risk of miscarriage.^49^ Today, all women are offered ‘combined’ screening for Down’s syndrome in the first trimester of pregnancy, with a quadruple screen available to some in the second.^50^ While many refuse it,^51^ 90% of those who accept screening and receive an antenatal diagnosis of Down’s syndrome go on to terminate their pregnancies.^52^ These developments have had a profound impact on debate regarding section 1(1)(d), which is no longer animated by concerns regarding ‘the slaughter of thousands of potentially healthy children to avoid the birth of a few deformed ones’.^53^ Rather, critics argue that routinely available testing may be accepted as a result of conformity, not active informed choice; that the information given may be skewed and inaccurate; and that the accuracy, safety and sophistication of screening and testing risks the creation of a ‘world without Down’s Syndrome’.^54^

This shift in the interpretation, implementation and criticism of the law also reflects a final important development since 1967: the rise of an active disability rights movement to challenge discriminatory, dehumanising and exclusionary attitudes. While progress has been uneven and remains incomplete, its campaigns have won important legal reforms, and have powerfully shaped public opinion and clinical practice.^55^ In 1993, the Royal College of Obstetricians and Gynaecologists (RCOG) welcomed the development of maternal serum testing with a confident prediction that the ‘new test could halve Down’s Syndrome Births’, implicitly assuming that the technology would be embraced by patients, that pregnancies known to be affected would be terminated and that this was an outcome to be celebrated.^56^ A very different sensibility is evident in the RCOG’s current clinical guidance, which emphasises that health professionals must adopt a non-directive, non-judgmental and supportive approach; that fully informed consent is paramount at all stages; that women’s decisions must be fully respected; and that no assumption should be made that a termination will be chosen even following the diagnosis of a fatal fetal anomaly.^57^

This brief biography of the Abortion Act offers vital context for *Crowter*, providing compelling evidence of the ‘obvious vice’ that the claimants identify with section 1(1)(d): that it reflects the outdated, discriminatory assumptions of the late 1960s. Written into the text of the Abortion Act is the belief that the ‘risk’ of a future child ‘suffer[ing]’ a ‘serious handicap’ offers an exception justifying the termination of a pregnancy. However, this biography also complicates this claim in two important ways. First, it recalls that current abortion law displays other, equally ‘obvious vices’. The Act is framed in a way that assumes maternity to be the natural desire and destiny of all healthy women and the normal outcome of any given pregnancy, with abortion permissible only in exceptional, state-sanctioned, professionally certified circumstances that warrant a departure from this norm. In giving two doctors the legal power to determine whether these conditions are met, the Act is marked by a powerful deference to medical authority, itself shaped by discriminatory gendered and class-based assumptions regarding the respective decision-making capacities of pregnant women and professional medical men.^58^ In attacking just one pillar of this flawed regulatory framework whilst leaving others intact, the arguments made on behalf of the *Crowter* claimants not only ignore these wider problems, but also pit the interests of disabled people against those of pregnant people, envisaging legal reforms to address disability oppression that would impact negatively on reproductive rights.

This account also complicates the claim that section 1(1)(d) is ‘in conflict with modern attitudes towards disabled persons’ in a second important way. While the Abortion Act’s text remains indelibly marked by the assumptions of the late 1960s, its implementation has evolved in line with changing social norms. There has been an increasing emphasis on professional responsibilities to offer non-directive, supportive care; to support informed decision making and respect patients’ choices; and to avoid discriminatory, reductive assumptions about what it means to live with a disability. This tension between the text of ageing legislation and its interpretation and implementation in modern clinical practice is of profound importance in *Crowter*.

## 3. Disability and Reproductive Rights in Opposition

### A. Previous Contestation Regarding Section 1(1)(d)

Section 1(1)(d) of the Abortion Act emerged as an important focus for anti-abortion groups in the early 1980s and within Parliament from the 1990s, with members of the Pro-Life All-Party Parliamentary Group (APPG) recommending measures designed to achieve the kinds of reforms now proposed in *Crowter*.^59^ In 2013, an APPG inquiry concluded that section 1(1)(d) is discriminatory and should be repealed or amended to include the upper time limit that applies to ‘able bodied babies’.^60^ This report was cited in support of two bills brought by Kevin Shinkwin, who was born with a brittle bone disease. Lord Shinkwin has been both a long-standing advocate for disability rights and guided by a ‘tireless … application of his theology to the cause of conservative philosophy’.^61^ The Shinkwin bills proposed similar reforms to those that the *Crowter* claimants would later describe as necessary in the courts: the first called for the repeal of section 1(1)(d);^62^ the second for its amendment to include a 24-week limit.^63^ He would later give evidence on their behalf.^64^

Section 1(1)(d) was not an immediate priority for a nascent disability rights movement, and the large and diverse modern movement encompasses a range of views on this (and other) issues. However, some disability rights organisations have been strongly critical of the provision. Most notably, in 2001, the newly formed Disability Rights Commission complained that the provision was ‘offensive to many people’, reinforced ‘negative stereotypes of disability’ and was ‘incompatible with valuing disability and non-disability equally’.^65^

A legal challenge soon followed from the Reverend Joanna Jepson, who had been born with a cleft palate and had a brother with Down’s syndrome. Jepson was selected to front the case by the veteran anti-abortion campaigner Josephine Quintavalle, who astutely predicted that she ‘would go down well in the papers’.^66^ She argued that a reported termination after 24 weeks following a diagnosis of cleft palate did not meet the requirement for a ‘serious handicap’ under the Abortion Act, and applied for judicial review of the decision not to charge the doctors involved.^67^ Framing her challenge within a ‘move towards a less discriminatory society’, Jepson posed a powerful rhetorical question later echoed by Heidi Crowter: ‘is society saying I should have died?’^68^ A further police investigation concluded that the doctors had acted in good faith and, therefore, lawfully.^69^ While the case provoked a ‘media frenzy’,^70^ with some apparent chilling effect on clinical practice,^71^ it did not proceed beyond an initial finding of legal standing. *Crowter* thus offered the first opportunity for a full judicial consideration of section 1(1)(d).

### B. *The Legal Arguments in* Crowter

In *Crowter*, a declaration was sought that section 1(1)(d) of the Abortion Act breached the claimants’ rights under articles 2, 3, 8 and 14 of the European Convention on Human Rights (ECHR); that section 1(1)(d) did not permit abortion on the basis of a non-fatal disability such as Down’s syndrome; and that it was unlawful for the government to provide funding so as to ‘promote the availability of abortion’ authorised under section 1(1)(d) (or at least those terminations authorised on the basis of a non-fatal disability such as Down’s syndrome).^72^ As such, echoing the proposals made in Parliament by the Pro-Life APPG and in the two Shinkwin bills,^73^ it was claimed that section 1(1)(d) should be ‘amended or repealed and/or significantly limited in its practical effect’.^74^

In support of these claims, it was argued that, first, an ‘unborn child’ capable of life outside the womb (and, in particular, in the period immediately before birth) fell within the category of ‘everyone’ to whom Convention rights must be afforded.^75^ Aidan Lea-Wilson had been ‘exposed to the risk of death by abortion when non-disabled children would not be so exposed’, breaching his right to life under article 2 ECHR and constituting discrimination under article 14.^76^ Further, it was claimed that he would have been sentient when a termination was offered and, as fetal anaesthesia is not mandated in abortion, the termination would have caused him ‘intense suffering’ in breach of article 3’s prohibition on torture, inhuman and degrading treatment.^77^ Moreover, given that article 3 protects against actions that violate dignity and physical integrity, it was engaged regardless of whether pain was caused, particularly where procedures involve physical dismemberment.^78^ These claims were robustly dismissed. The Divisional Court noted that the European Court of Human Rights had never found a fetus (including post-viability) to be the bearer of Convention rights, leaving ‘the controversial and difficult issue of when life begins’ within the margin of appreciation enjoyed by contracting states.^79^ While the state was entitled to protect fetal interests, this did not mean that a fetus enjoyed Convention rights.^80^

The more plausible legal arguments for the claimants lay under article 8, again joined with article 14, where two distinct claims were advanced. First, it was argued that section 1(1)(d) ‘perpetuates and reinforces negative cultural stereotypes to the detriment of people with disabilities’,^81^ resulting in ‘a serious diminution in the perception of the value of [Heidi Crowter and Aiden Lea-Wilson’s] lives’, discriminating against them in their enjoyment of rights to dignity, autonomy and personal life.^82^ It was noted that the UN Committee on the Rights of Persons with Disabilities (CRPD Committee) has criticised laws that single out fetal anomaly as a specific reason for abortion. Further, an expert witness gave evidence that ‘institutional stigma, such as that inherent in legislation, has a powerful role to play in either countering or promoting and maintaining negative stereotypes or discrimination’ and that, in her view, section 1(1)(d) ‘powerfully communicates a message that the lives of persons with conditions such as Down Syndrome are “not worth living”‘.^83^

Whilst arguing that it was sufficient to show that their sense of identity and self-worth was negatively impacted,^84^ the claimants also offered a very concrete example of the harm caused by section 1(1)(d): the ‘devastating’ impact of a storyline in the soap opera *Emmerdale* following a couple’s decision to terminate a pregnancy after a diagnosis of Down’s syndrome. This had led to ‘a very public debate on the validity of the existence of people with Down’s syndrome and their perceived value in society’, a slew of hurtful, discriminatory comments in social and traditional media, and some individuals reporting abuse.^85^ Máire Lea-Wilson explained that the story was broadcast only because of the discriminatory law, which had ‘an important role in forming people’s views of what is right and wrong’ and in normalising ‘a culture of discrimination which for people like my son begins even before they are born’.^86^

A second claim under articles 8 and 14 was made on behalf of Máire Lea-Wilson, who described being offered the option of termination three times following a late diagnosis of Down’s syndrome. She had opted out of earlier testing but, following an ultrasound scan at 34 weeks, a ‘brusque’ doctor had made a possible diagnosis of Down’s syndrome and suggested NIPT to confirm it, leaving Lea-Wilson crying so hard that her husband needed to sign the consent forms.^87^ Whilst waiting for the NIPT results, she saw a ‘very sombre’ obstetrician, who again explained the diagnosis and asked what they ‘would like to do next’, offering amniocentesis as a quicker way of confirming the diagnosis, allowing more time if they decided to terminate the pregnancy.^88^ Lea-Wilson recognised that the doctor thought that she was ‘being kind’ in ‘laying our options out so we could make an informed choice’, but complained that she had emphasised negative, medical aspects of Down’s syndrome and provided no ‘information on the lived experience’.^89^ The offer of abortion ‘during a time of great vulnerability and fear, with such a heavy bias and unbalanced information’ meant that she and her husband felt ‘steered towards’ and ‘forced to consider’ abortion, making them question their ability to cope and contributing to their ‘fear of having a child with Down’s Syndrome in a very real way’.^90^ Subsequently, her NIPT results were confirmed in a telephone call that began ‘It’s bad news I’m afraid’, with the caller asking if she needed to talk to someone about ‘next steps’ and—when told that she was intending to continue the pregnancy—replying that she was ‘inspirational’.^91^ At her next appointment, a different doctor again asked if she wished to continue her pregnancy.^92^ Only then did she see her named obstetrician, who had returned from leave. He told her that she was going to have a ‘very special little boy’, becoming the only medical professional who described her ‘son as a baby not pregnancy, and not as bad news’.^93^

A survey of mothers of children born with Down’s syndrome in the UK between 2000 and 2018 was cited to demonstrate that Lea-Wilson’s experiences were far from unique.^94^ Many of the 1410 respondents reported that prenatal testing was presented as routine rather than optional; described an expectation that those with a higher chance of Down’s syndrome would take tests and terminate an affected pregnancy; and had experienced positive diagnoses being presented as ‘bad news’. Some reported advice that termination was the ‘kindest thing to do’, and described struggling to challenge doctors who repeatedly offered abortion.^95^ Lea-Wilson argued that these experiences were directly caused by section 1(1)(d), which exposed some to ‘pressure to terminate their pregnancy all the way up to birth (whereas other women, with apparently healthy fetuses, will not come under any such pressure, or not after 24 weeks)’.^96^

The Divisional Court also dismissed both claims under articles 8 and 14, reasoning that section 1(1)(d) was concerned with ‘the rights of pregnant women’ and that any discrimination occurred despite extensive legislative provisions aimed at preventing it.^97^ Article 8 protected the decision to become a parent but an individual case could not be used to suggest that primary legislation was incompatible with it, and the treatment described by Máire Lea-Wilson was contrary to clear professional guidance.^98^ Moreover, any interference with article 8 rights would have been in accordance with the law and justified by and proportionate to the legitimate aim of protecting the rights of women.^99^ Article 14 was thus not engaged. However, even if it were, there was an objective and reasonable justification for any differential treatment, with states allowed a wide margin of appreciation and Parliament better placed to weigh ‘the intensely difficult issues’ involved.^100^

The other remedies sought were also denied. The Court recognised that most people diagnosed with Down’s syndrome would enjoy a life expectancy of 50–60 years and a good quality of life; however, a sizeable minority of pregnancies would result in stillbirth (2.6–5.4%) or death in childhood (16.6%), with those children that survived experiencing symptoms that varied greatly in severity.^101^ It declined to rule on whether this was sufficient to amount to a ‘serious handicap’, a finding that would ‘amount to impermissible judicial legislation and not interpretation’, going against the grain of the Abortion Act.^102^ It also rejected the request for a declaration that the government should stop funding abortions performed under section 1(1)(d): even if a breach of human rights were established, the only available remedy was a declaration of incompatibility.^103^

Having lost in the Divisional Court, Crowter and Aiden Lea-Wilson won permission to appeal only regarding the impact of section 1(1)(d) on living disabled people (ie not on the basis of asserting fetal rights), with claims therefore centred on articles 8 and 14 only. In the Court of Appeal, their lawyers argued, first, that section 1(1)(d) offered an inherent insult to identity and human dignity, and second, that it served to promote societal attitudes that caused discriminatory behaviour by third parties.^104^ An interference with article 8 might be established merely on the basis of negative stereotyping capable of impacting on disabled people’s sense of identity and feelings of self-worth and self-confidence, thereby affecting their private lives.^105^ Such interference could not be justified as ‘in accordance with the law’ and ‘necessary in a democratic society’ under article 8(2). First, section 1(1)(d) was disproportionate: it allowed abortion until birth for fetal anomalies that were correctable, were compatible with a happy and fulfilled life, manifested only later in life or were detectable early in pregnancy. Second, it was impermissibly vague: its broad framing allowed divergent interpretations by individual doctors acting without adequate judicial oversight.^106^ The Divisional Court had wrongly concluded its analysis by finding it impossible to provide a precise test for ‘serious handicap’, failing to give due consideration to the possibility of a test that was ‘as precise as practicable in all the circumstances’, perhaps taking the form of an exclusionary list, *inter alia* ruling out abortion on the grounds of a diagnosis of Down’s syndrome. Finally, the Divisional Court had overestimated the breadth of the state’s margin of appreciation, attaching insufficient significance to the ‘very weighty reasons’ necessary to justify discriminatory measures.^107^

With nothing in the jurisprudence of the European Court to suggest that a fetus could enjoy Convention rights, the Court of Appeal focused exclusively on the rights of the ‘living disabled’. It noted that section 1(1)(d) was not concerned with this group, thereby distinguishing the case before it from existing article 8 case law that focused on negative stereotyping of groups to which claimants belonged.^108^ While section (1)(d) might reflect long-established prejudices, that did not demonstrate that it perpetuated them.^109^ Some disabled people were upset and offended, perceiving the provision to imply that their own lives were of lesser value; however, others felt differently or were entirely unaware of it.^110^ The impact of an offending message must surpass a threshold of seriousness that, all things considered, had not been met here.^111^ Further, the existence of a legal right could not depend solely on the subjective perception of the victim: an interference with article 8 rights required that a message is unequivocally conveyed on the basis of an objective standard.^112^ Section 1(1)(d) reflected a balance, struck by Parliament in an area where it enjoyed a wide margin of appreciation, between the rights of pregnant women and the interests of the unborn.^113^ Decisions made by doctors necessarily involve complex professional judgments of a kind that do not lend themselves to specific statutory guidance.^114^ The one female judge noted that the decision to end a pregnancy was ‘the right and personal responsibility of the woman, in accordance with the law’, that she was ‘uniquely placed to make it’ and that this did not ‘have the effect of stigmatising the living disabled’.^115^

On this basis, the Court dismissed the appeal. Crowter reported that she was ‘absolutely distraught’.^116^ With permission to appeal to the Supreme Court subsequently denied, at the time of writing her lawyers are applying to proceed to the European Court of Human Rights.^117^

### C. *The Anti-abortion Framing of the Legal Arguments*

The biography of any law is importantly shaped by the biographies and commitments of a diverse, shifting cast of actors. As briefly seen above and described in more detail elsewhere, campaigners have played an important role in bringing and supporting many of the cases regarding the Abortion Act.^118^ Notwithstanding the often-repeated claim that they apply the law ‘free of emotion or predilection’, judges too have given meaning to the law in a way that is shaped more or less explicitly by their moral or religious beliefs and life experiences.^119^ The importance of the biographies of the solicitors and barristers involved have been less widely recognised. Whilst their names are listed law reports, it is rare that they receive any mention in the text of a judgment beyond, perhaps, a note of thanks for their clarity of exposition on a specific point. However, lawyers play a vital role in determining which claims are worth pursuing, what remedies might be sought, and what arguments should be put to the court and how they should best be framed, a fact obscured in the conventional synecdoche of writing ‘the claimant argued’ rather than the wordier but more accurate ‘the claimants’ lawyers argued on their behalf’. The biography of the Abortion Act has been clearly marked by the work of many lawyers over the past five decades, with a powerful influence on how the Act should be interpreted and how challenges to (and defence of) it should be framed.^120^

In *Crowter*, the claimants’ legal team was led by Jason Coppel KC, a leading expert in the broad fields of public law, procurement law, information law and human rights. Coppel has acted in at least three previous abortion cases (twice representing the UK government and once the Equality and Human Rights Commission), but has no particular track record of advocating for restricting access to abortion, or representing campaigning organisations with this aim.^121^ However, his junior was Bruno Quintavalle, who co-founded the ProLife Alliance with his mother, Josephine (who, 10 years earlier, had selected Joanna Jepson to front the previous challenge to section 1(1)(d)); and the advising solicitor was Paul Conrathe, who had also represented Jepson.^122^ Quintavalle and Conrathe each specialise in cases of concern to Christian groups,^123^ with Conrathe acting in a large number of previous cases that had sought piecemeal restrictive reform of the regulation of abortion. *Inter alia*, Conrathe represented Sue Axon, who fought for the right for a parent to be informed before a minor child is permitted to terminate a pregnancy;^124^ Stephen Hone, who sought to prevent his former partner from terminating her pregnancy;^125^ and Ann-Marie Tudor, who attacked NICE guidelines for their failure to require that women be informed that a fetus may feel pain.^126^

It is not uncommon for lawyers’ biographies to suggest a commitment to using their legal skills to advance particular political, moral or religious causes: individual lawyers often develop areas of professional expertise that reflect personal interests and moral and political values. This is no impediment to effective, ethical professional conduct.^127^ Indeed, clients may seek out a lawyer precisely because of this reputation, with restrictions in the availability of legal aid meaning that all but the wealthiest can struggle to find an appropriately skilled lawyer prepared to bring a complex judicial review action on their behalf. However, these biographies are nonetheless relevant to how the harms described by the claimants were likely to be understood from within the ‘tram lines’ of current, deeply polarised abortion debates shaped, *inter alia*, by religious faith; and they were inevitably influential in decisions regarding how legal arguments should be framed, with the remedies sought fitting neatly into a wider strategy of ‘chipping away’ at the current, ageing abortion law to achieve its piecemeal, restrictive reform.

On the other side, the government was represented by Sir James Eadie KC, a leading public and regulatory law specialist and ‘Treasury Devil’, supported by two juniors who specialise in public and human rights law and who had acted for the government in earlier abortion cases.^128^ In responding to the arguments made by the claimants’ lawyers, the government’s legal team necessarily focused on refuting claims for restrictive reform of the Abortion Act. Whilst briefly noting the potentially ‘devastating’ consequences for pregnant women and their families of legislating in the way contended for by the claimants’ lawyers, they emphasised that a fetus cannot enjoy human rights, that it was for Parliament to reach a decision in such an ethically complex area and that the Abortion Act had been passed only following extensive and rigorous debate.^129^ No intervention was made on behalf of pro-choice groups or women’s organisations, and limited reference was made to women’s rights in argument.

Any court action involves the translation of broader moral and political grievances into legal claims and demands for specific remedies, selected partly with an eye on what is most likely to succeed in the courts (or outside them, with litigation also sometimes conducted with an eye to shaping public opinion). Further, if a Declaration of Incompatibility had been won in *Crowter*, the impact would have been to force consideration by Parliament, which might then choose to react in a range of ways. Nonetheless, a victory in *Crowter* would have sent a clear message regarding the specific problem to be addressed in any resulting reform of the law. I argue that the arguments made for the claimants—and the kinds of legal reform envisaged in the action—are best understood as part of a wider anti-abortion strategy, to be achieved through piecemeal restrictions on access to abortion services, with the kinds of reforms envisaged echoing the restrictive statutory reforms earlier proposed by the Pro-Life APPG and Lord Shinkwin.^130^ In what follows, I revisit the arguments presented to the courts in order to flesh out this claim. Further, I argue that the specific legal claims made in *Crowter* were not inevitable, with other avenues available for seeking to address the social harms described by the claimants. Indeed, I argue that the kinds of legal reforms envisaged in the action—focusing on the repeal or removal of section 1(1)(d)—were at once disproportionate and insufficient to address those harms.

How is a wider anti-abortion framing visible in the legal arguments? First, the claim that human rights are enjoyed *in utero* reflects a foundational belief for many anti-abortion campaigners, often grounded in religious faith: that ‘unborn children’ enjoy the same moral rights—and deserve the same legal protections—as those born. Claims about disability discrimination were thus framed as claims about discrimination against unborn persons *in utero*. On behalf of the *Crowter* claimants, it was argued that viability is the ‘only logical and legally sensible test’ for determining the moment when such rights are acquired, with it making ‘far less sense to distinguish between [Aiden Lea-Wilson] whilst he was in the womb and, a few minutes later, after he had been surgically, and prematurely, removed from the womb’.^131^ The logic of this claim derives from its exclusive focus on the fetus, ignoring the profoundly meaningful difference from the perspective of a pregnant (or no longer pregnant) person, with the former having a visceral, intimate and unique connection to a fetus *in utero*. Moreover, sitting in some tension with the statement that viability offers ‘the only’ sensible test in this context, elsewhere the claimants’ lawyers take care to leave open the possibility that human rights may be achieved earlier, being enjoyed ‘at least’ at the point of viability and ‘in particular’ in the period immediately before birth.^132^

A finding that a fetus holds rights under the ECHR would have represented a seismic change in current law, with implications potentially extending far beyond the parameters of the current action. Just one such consequence was addressed: it was argued that this finding would not preclude abortion where necessary to save the life or preserve the health of the pregnant woman, citing *Re A (Conjoined Twins)*.^133^ However, even if a case on such different facts were accepted as a relevant precedent, *Re A* would appear likely to permit termination only in far more limited conditions than the current statutory grounds for abortion (or, indeed, the previous common law test).^134^ More generally, no consideration was given to the wide-ranging potential consequences of a finding that rights can be held *in utero*, notwithstanding the clear and troubling precedents to be found in other jurisdictions.^135^

Second, the wider anti-abortion framing of the legal arguments is visible in the assumption that banning abortion is a necessary and proportionate response to a concern that later abortion might cause pain to the fetus (an empirical claim subject to significant ongoing debate).^136^ The claimants relied on expert testimony from Professor John Wyatt (Emeritus Professor of Neonatal Paediatrics, University College London, Chair of Kirby Laing Institute for Christian Ethics and President of the Christian Medical Fellowship), who highlighted an important inconsistency in practice whereby fetal anaesthesia is not mandated in abortions whilst being routinely used in fetal surgery after 18 weeks.^137^ In light of such concerns and the potential anxiety thereby caused to women and health professionals, the British Medical Association has recently recommended that fetal anaesthesia should be used in abortions performed after 18 weeks, notwithstanding lack of definitive proof of fetal sentience.^138^ Ignoring this potential response, the claimants’ lawyers rather argued for a ban on abortion after 24 weeks (which would have served not to eliminate any inconsistency, but rather to limit its scope to abortions performed at 18–24 weeks’ gestation). Indeed, the possibility that a concern with fetal sentience might be addressed through mandating the use of anaesthesia is implicitly refuted by the additional claim that a later abortion procedure—and particularly a surgical one—would breach article 3 independently of whether it causes pain. If successful, this claim would again potentially have had wide purchase, logically extending to all later abortion procedures (or at least all surgical ones), including when performed to prevent grave permanent injury to the pregnant woman’s health or avert risk to her life.^139^ Article 3 does not admit exceptions where a *prima facie* breach may be justified as necessary with reference to other considerations.

Third, it is easy to imagine responses to Máire Lea-Wilson’s experience that do not depend on restricting abortion law, including: a complaint to the hospital where she was treated that professional guidance had not been followed; demands for improvements in informed consent procedures and further training of health professionals; and proposals for the development of specialist antenatal care pathways and greater continuity of care for women in her situation. In *Crowter*, however, it is argued that Lea-Wilson was ‘forced to consider’ abortion simply by virtue of the fact the option was legally available to her, with similar pressure avoidable only by removing this option from all women.

In this respect, the remedies pursued in *Crowter* diverge starkly from recommendations contained within reports on which the claimants’ lawyers themselves relied, with the reports avoiding claims for fetal personhood. The PADS report was cited as evidence of widespread problems in antenatal care, with PADS’s founder, Nicola Enoch, giving evidence that change would only happen when ‘a baby with [Down’s syndrome … is] afforded the same rights in the womb as any other baby’.^140^ However, the report itself eschews advocating for restrictive abortion law reform in favour of calling for a series of practical changes that tend largely to support a more robust and consistent implementation of existing RCOG guidance.^141^ A report from MENCAP relied upon in evidence likewise conspicuously refrains from recommending restrictions on access to abortion (or in the use of NIPT) in favour of recommending a series of changes to policy and practice informed by ongoing consultation with people with Down’s syndrome.^142^ This report was informed by six in-depth interviews with people with Down’s syndrome who themselves expressed careful and nuanced views: they generally agreed with prenatal testing as a means to prepare prospective parents and were saddened when it resulted in termination, but nonetheless generally respected women’s right to choose.^143^

The apparent paradox in arguing that women’s reproductive rights should be limited as a means of protecting and promoting women’s interests reflects a familiar wider ‘woman-protective’ turn in anti-abortion campaigning, with any paradox evaporating when abortion is understood as intrinsically harmful to women, actively encouraged by a biased ‘abortion industry’ and never voluntarily chosen by women given objective information, appropriate support and adequate time to reflect.^144^ The framing of a demand for an end to public funding to ‘promote’ abortions authorised under section 1(1)(d) likewise implicitly reflects a belief that abortion is actively encouraged rather than made safely and legally available for those who actively choose it. The demand also ignores the gross social inequality that would potentially again result (and which in the 1960s had been a major driver of abortion law reform^145^) were the wealthy able to terminate unwanted pregnancies while others had maternity enforced by an inability to afford an abortion.

Fourth, the claimants’ lawyers are right to identify the anachronistic and discriminatory assumptions reflected in the framing of section 1(1)(d) and to note that they accord poorly with modern attitudes towards disabled people. While inevitably challenging to prove that the provision directly causes stigma and other harms, they offer compelling evidence that its continued existence is a matter of distress to some.^146^ Moreover, it is true that some disability rights organisations—most importantly, the CRPD Committee—condemn laws that exceptionalise abortion on the grounds of fetal anomaly.^147^ However, refracted through an anti-abortion lens, these important concerns are translated into a call for restrictive reform seeking the removal of just one provision of the anachronistic, flawed statutory framework. The claim that current law leaves too much to the discretion of individual doctors is marshalled as part of an attack narrowly focused on section 1(1)(d), ignoring that the entire statutory framework is marked by a deference to clinical discretion and implicit medical paternalism (a position that aligns equally poorly with contemporary social norms foregrounding the primacy of women’s wishes). Further, as discussed below, while the CRPD has called specifically for the repeal of section 1(1)(d), its criticism of selective abortion forms just one part of a broader critique of disability discrimination; and its recommendations for law reform are carefully framed to take account of reproductive rights.

Fifth, the biographies of litigants are also important. Any legal action necessarily focuses on a particular individual (or individuals), sometimes specifically chosen with an eye to their chances of success within the courts or in shaping opinion outside them (as was the case in *Jepson*).^148^ In support of a call for an end to all post-viability terminations (other than possibly for fatal fetal anomalies), *Crowter* focuses on the experience of two happy and healthy disabled people with a high quality of life and the active support of loving families. However, Down’s syndrome is a spectrum disorder, and sadly some will experience more severe, life-limiting and life-threatening symptoms.^149^ Likewise, the criticisms made of antenatal care focus on the experience of Máire Lea-Wilson, who was offered an abortion after diagnosis of Down’s syndrome at 34 weeks. While evidence from PADS suggests that some aspects of Lea-Wilson’s experiences were all too common, other aspects were vanishingly rare. In 2019, 207,384 abortions were reported for women resident in England and Wales, with 275 of them performed under section 1(1)(d) after 24 weeks. Just 13 of these were on the basis of a diagnosis of Down’s syndrome alone (with it mentioned in conjunction with another medical condition in a further six cases), and none occurred at the very late stage that a termination was offered to Lea-Wilson.^150^ This matters: beyond the *Crowter* claimants lie a range of individuals struggling to process a variety of complex antenatal diagnoses for conditions that vary greatly in severity, and where it is often impossible to predict what a specific diagnosis will mean for a particular future child.^151^ The repeal or restriction of section 1(1)(d) would limit access to abortion, and remove the possibility for these individuals to make decisions in consultation with their doctors. It would force, on pain of onerous possible criminal sanction, the continuation of pregnancies that would have been unwanted by some in this group; curtail decision-making time and the possibility of full information from all available tests for others; and place health professionals in the invidious situation of ‘effectively relegating [their] job of properly caring for women in difficult circumstances and allowing them time to decide what is right for them’.^152^

Finally, *Crowter* reduces important moral and political concerns with disability discrimination into a narrowly framed attack on one potential manifestation of it, ignoring root causes. The arguments made on behalf of the claimants emphasised the need to repeal or reform section 1(1)(d) or to require its more restrictive interpretation, measures that are not just disproportionate, but also woefully inadequate to address the moral and social harms that they described. The request that section 1(1)(d) be subject to the same upper time limit as most other abortions is logical within a pragmatic anti-abortion philosophy that celebrates every diminution in the number of abortions in terms of lives saved. It makes less sense as a means of addressing the discriminatory and hurtful message expressed in an outdated statutory provision that recognises ‘substantial risk’ of ‘suffering’ ‘serious handicap’ as an exceptional and acceptable reason for refusing motherhood. If the concern is with the message conveyed, then writing a time limit into section 1(1)(d) offers, at best, a partial and inadequate resolution. The concern would be more fully addressed by the repeal of section 1(1)(d); however, that would not affect access to abortion on grounds of the risk to women’s mental and physical health, which has been subject to a wide interpretation.^153^ Doctors might lawfully continue to authorise abortion on that basis to an upper time limit of 24 weeks, permitting the vast majority of abortions currently performed under section 1(1)(d) to continue (and, indeed, potentially increasing their number if decisions are rushed to meet that deadline, without the benefit of all tests and adequate reflection time).

Neither would the remedies sought in *Crowter* directly affect current prenatal screening and testing practices, which many disability rights advocates have identified as a more pressing concern.^154^ A future soap opera storyline might unfold in the same way as that complained of in *Emmerdale*, potentially provoking the same hurtful and discriminatory comments. No progress would be made towards addressing the important PADS or MENCAP recommendations for improvements in information and support for those facing a prenatal diagnosis of disability in a future child, nor in the training of the staff who provide it, nor in the involvement of disabled people in the design of antenatal care pathways. Indeed, imposing a gestational upper limit on section 1(1)(d) would necessarily cut against some recommendations, reducing the time available for careful reflection and for patients who so wish to contact local support groups or families that include someone with the relevant disability.

Finally, it was noted that the CRPD Committee has called for the repeal of legal provisions—such as section 1(1)(d)—that exceptionalise abortion for fetal anomaly, on the basis that they risk reinforcing and validating the message that persons with disabilities ought not to have been born.^155^ However, it is important to understand this recommendation in its wider context. In 2018, the CRPD Committee joined with the United Nations Council for the Elimination of Discrimination Against Women to issue a Joint Statement on gender equality and disability rights, arguing that these should be understood not as conflicting, but rather as ‘mutually reinforcing concepts’.^156^ While recommending the repeal of abortion laws that perpetuate stereotypes, the Joint Statement proposes a very different model of reform: the decriminalisation of abortion in all circumstances and the legal provision of abortion services in a manner that fully respects the autonomy of all women, including those with disabilities.^157^

## 4. Disability and Reproductive Rights as ‘Mutually Reinforcing Concepts’

The Joint Statement offers a very different perspective from which to consider the important moral and political concerns raised by Heidi Crowter and Máire Lea-Wilson. It locates the removal of offending and discriminatory legal provisions as an important but nonetheless small part of a much wider social and political project, highlighting the rights of a group whose sexual and reproductive rights have often been most egregiously ignored and infringed: women with disabilities.^158^ It also takes a broader view of state responsibilities that extend far beyond abortion law and antenatal care to encompass the conditions in which reproductive choices are made, bringing into focus the need for adequate housing, inclusive education and employment, and sufficient and respectful social welfare provision.

These factors should all be of profound importance to those who care about both reproductive and disability rights: unless proper account is taken of the social structures that support all parents, disabled people and their families and carers, we ignore the conditions that make reproductive choice meaningful and thereby risk shifting responsibility from social systems onto individuals and calling it patient autonomy.^159^ As well as individual circumstances, the personal decision (not) to continue a pregnancy will reflect the options available; the way that those options are presented; the wider legislative and policy frameworks that make living with a disability—or caring for a disabled child—more or less challenging; and wider social norms that may reflect inaccurate, discriminatory assumptions. Moreover, when considered collectively, individual decisions may combine to have powerful and wide-ranging social consequences.^160^ This means that it is simultaneously true yet also inadequate to respond to claims of disability discrimination—as did Thirlwall LJ in the Court of Appeal—by noting that abortion decisions are intensely personal ones, taken in the context of individual circumstances and not to be read as dismissing the value of disabled people’s lives.^161^

The demands on the health professionals who work in this area are considerable and can only be exacerbated by the need to operate in the shadow of a stigmatising criminal law framework. Professional guidance that emphasises the need for non-directive, non-judgmental, supportive practice is necessary but insufficient, and the challenges involved in fully and consistently implementing it should not be underestimated. Detailed knowledge, considerable skill and ‘serious emotional labour’ are required to offer clinically accurate information in a way that facilitates genuine informed choices by diverse patients characterised by individual needs, expectations and beliefs, and grappling with a wide range of diagnoses.^162^ Health professionals are called to walk a line so fine as to be near invisible between ensuring that their patients are fully aware of all options available to them and able to exercise meaningful choice whilst simultaneously avoiding displaying moral judgment of any decision that might be made, consciously or unconsciously exerting pressure to follow a particular course of action, stigmatising patients or heightening their anxiety as they struggle to come to terms with complex medical information. Obstetricians are frequently aware of the limitations in their own knowledge of a particular condition, yet must endeavour to explain it in a way that does not reduce a future child to nothing more than a medical diagnosis; that neither denies nor exaggerates the joys and challenges of raising a child with a particular condition; and that avoids exceptionalising disability by forgetting that all parenting is demanding work. Further, doctors must acknowledge the limitations of existing diagnostic tools, which may sometimes accurately predict the presence of a particular syndrome with a high level of certainty, yet say little about its severity in a future child and their likely abilities, disabilities and quality of life.^163^ Finally, it is not only doctors but the range of health professionals with whom women interact in the antenatal care pathway whose words and actions shape experience of care.

Máire Lea-Wilson’s testimony powerfully exemplifies the challenges involved in getting this highly skilled work right and the potential pain and anxiety caused by getting it wrong. The best-intentioned doctor may believe herself to be offering an option only for this to be heard as a recommendation. Clumsy attempts to offer validation and support (such as praising a decision to continue a pregnancy as ‘inspirational’) can convey a frightening and offensive message that a decision to parent a child with a specific disability reflects exceptional bravery or self-sacrifice. Sometimes—as in choosing whether to refer to a ‘baby’ or a ‘pregnancy’^164^—there is no one terminology likely to respond to the emotional needs of all patients. Even where seemingly neutral language is available (for example, describing the ‘probability’ rather than the ‘risk’ that a child will ‘have’ rather than ‘suffer from’ a particular condition), training must go beyond the avoidance of certain words to unpack, acknowledge and fully address the unconscious assumptions that underpin our linguistic choices. This is inevitably more challenging and requires ongoing work not just from health professionals, but from all of us.^165^

Whilst the challenges are thus considerable, the Joint Statement’s call to treat the rights of women and disabled people as ‘mutually reinforcing concepts’ offers a better starting point for navigating these complex and important concerns. When the eminent disability studies scholar Professor Tom Shakespeare argues for information to be offered in a way that conveys something ‘about the rich and varied lives of disabled people, not just … about genetic spelling mistakes’, he is demanding not just that the valuable, complex and multifaceted lives of disabled people must not be reduced to a particular condition; but also that we must respect the autonomy of those who rely on that information to make profoundly important decisions about their future parenting.^166^ The repeated offer of a termination to a woman who has clearly communicated an intention to continue her pregnancy offends against a woman’s reproductive rights as well as conveying a discriminatory message about the value of disabled life. There is fertile common ground between the recommendations made by disability rights groups, professional medical bodies and reproductive rights organisations for measures that aim to ensure that ‘informed choice’ is a richly meaningful process rather than an empty slogan.

Reform of abortion laws is a small but nonetheless important part of this wider political agenda. Here, the Joint Statement avoids demanding respect for disability rights via the restriction of reproductive rights, instead calling for decriminalisation of abortion. This call is echoed in authoritative guidelines, published by the World Health Organisation, that recommend full decriminalisation (including the removal of gestational limits and specific grounds) to make abortion available on request, within a broader ‘enabling environment’ of quality comprehensive abortion care, including support for continued pregnancy and parenting.^167^

Depending on the precise framing of any legislation, decriminalisation might offer a productive first step towards addressing at least some of the concerns raised by Heidi Crowter and Máire Lea-Wilson.^168^ It could remove the offensive statutory language of ‘handicap’, ‘risk’ and ‘suffering’ contained in the Abortion Act.^169^ Indeed, it would be likely to offer a wholescale dismantling of the exceptions-based statutory framework, with its underlying assumption that the avoidance of the birth of a disabled child offers a legitimate, acceptable reason for ending a pregnancy while an individual’s many other possible motivations for refusing parenthood at a particular time do not. With a focus on reproductive and disability rights, debate of a new law might also offer the opportunity to broaden discussion to include consideration of whether additional statutory safeguards might help to ensure full respect for the sexual and reproductive rights of disabled women.^170^ The extent to which any new law might achieve these goals necessarily depends on detail that there is no space here to explore. It should be noted, however, that those jurisdictions which have decriminalised abortion have not seen a resulting increase in the absolute number of abortions, nor in abortions occurring later in pregnancy.^171^

In sum, while there are no easy answers to the complex problems raised in *Crowter*, it has been argued that there are better places to search for them, and that these are not readily visible from within the ‘tram lines’ of current abortion debates. We should not start from the assumption—apparently shared by very few modern British adults—that a fetus is a person, capable of enjoying human rights and requiring protection from discrimination in its own right.^172^ However, the broad moral and political claims advanced by Crowter and Lea-Wilson outside the courts are capable of commanding much wider support. Heidi Crowter was right to insist that she be ‘seen as equal in society’.^173^ Máire Lea-Wilson should be supported in her hopes for

new and expectant parents … to be given fair and unbiased information about [Down’s syndrome] and, above all else, that … people like my son start to look to a future where they can live a life free from discrimination.^174^

These are important goals for those who care about reproductive as well as disability rights, offering a further argument for abortion law reform that better reflects modern moral values.

I have argued that subsequent steps towards such laws would be better taken from a position that views reproductive and disability rights as mutually reinforcing, offering a basis for a shared rejection of anachronistic gender norms, medical paternalism and discriminatory assumptions about disability. For reproductive rights advocates, this requires taking seriously the concerns raised in *Crowter* and building new alliances in seeking to address them. It also involves moving outside the ‘tram lines’ of existing debate and beyond the politics of piecemeal reform of existing flawed and anachronistic legislation.

## Footnotes

1 John Pugh, HC Deb 6 July 2015, vol 598, col 109.

2 David Alton, ‘Saving the Alton Bill’ *The Times* (London, 21 May 1988).

3 David Alton, *What Kind of Country?* (Marshall Pickering 1988) 170.

4 See Simon Hattenstone, ‘‘My Life is Just as Important as Everybody Else’s”: Meet the Disability Leaders’ (London, *Guardian,* 3 December 2018). Crowter married during the court action and is now Heidi Carter, but, for ease, I refer to her throughout by the name used to bring the case. I use the term ‘anomaly’ to describe an abnormality in a fetus that implies a likely impairment or disability in a future child; ‘impairment’ to describe a physical or mental abnormality in a living person; and ‘disability’ or ‘disabled’ person to recognise that disadvantage suffered as a result reflects interaction between impairment and a wider environmental and social context, whilst remaining vigilant to the risk that universal tags can obscure the enormous diversity of disabled people’s lives. I follow the standard UK English convention of referring to Down’s syndrome, notwithstanding the criticisms that have been made of the possessive form, see David Wright, *Downs: The History of a Disability* (OUP 2011).

5 Claim Form, in Core Bundle, submitted to the Divisional Court in Case No: CO/2066/2020, on file with the author.

6 Máire Lea-Wilson, cited in CNA staff, ‘Meet the Mother Challenging the UK’s Down Syndrome Abortion Law’ (Catholic News Agency, 20 May 2021).

7 Don’t Screen Us Out, ‘Woman with Down’s Syndrome’s Landmark Case against UK Govt over Discriminatory Abortion Law to Be Heard by High Court’ (Press Release 18 October 2020) <https://dontscreenusout.org/press-release-woman-with-downs-syndrome-landmark-case-against-uk-govt-over-discriminatory-abortion-law-to-be-heard-by-high-court/> accessed 1 June 2023.

8 Sally Sheldon and others, *The Abortion Act 1967: A Biography of a UK Law* (CUP 2022).

9 Carol Harlow and Richard Rawlings, *Pressure Through Law* (Routledge 2016).

10 ibid; Sheldon and others (n 8).

11 ibid.

12 For a full list of archives consulted, see Sheldon and others (n 8) 306–7.

13 On this use of ‘biography’, see generally ibid 18–19, Sally Sheldon and others, ‘The Abortion Act 1967: a Biography’ (2019) 39(1) LS 18.

14 Statement of Facts and Grounds in Core Bundle (n 5) [35].

15 For a brief reflection on the significance of the biographies of some of the academics who have written on the Abortion Act, including my own, see Sheldon and others (n 8) 24–6.

16 Sally Sheldon, ‘The Decriminalisation of Abortion: An Argument for Modernisation’ (2016) 36 OJLS 334.

17 The 1861 Act applies in England and Wales. A second, overlapping offence is provided by the Infant Life (Preservation) Act 1929. In Scotland, the offences are to be found in common law. See generally ibid.

18 This wording reflects amendments made in 1990, when the Act was reformed to provide for termination without time limit on the grounds of fetal anomaly. For a detailed discussion of these changes, see Sheldon and others (n 8) 100–6.

19 On this wider history, see ibid; Keith Hindell and Madeleine Simms, *Abortion Law Reformed* (Peter Owen 1971); John Keown, *Abortion, Doctors and the Law: Some Aspects of the Legal Regulation of Abortion in England from 1803 to 1982* (CUP 1988); Sally Sheldon, *Beyond Control: Medical Power and Abortion Law* (Pluto Press 1997).

20 Lesley Reagan, *Dangerous Pregnancies: Mothers, Disabilities and Abortion in Modern America* (University of California Press 2010); Hindell and Simms (n 19).

21 Hindell and Simms (n 19) 87.

22 Reagan (n 20) 104.

23 Glanville Williams, *Textbook of Criminal Law* (2nd edn, Stevens 1983) 297. Williams was a Cambridge law professor, who drafted the Abortion Act.

24 Renée Short, HC Deb 22 July 1966, vol 732, col 1162.

25 Gareth M Thomas, *Down’s Syndrome Screening and Reproductive Politics: Care, Choice, and Disability in the Prenatal Clinic* (Routledge 2017) 31. See generally Wright (n 4).

26 Reagan (n 20).

27 Norman St John-Stevas, HC Deb 29 June 1967, vol 749, col 1050.

28 For a rare exception, see Kevin McNamara, HC Deb 22 July 1966, vol 732, col 1128, citing a letter by three residents of the Thomas Delarue School for Spastics.

29 See generally Sheldon and others (n 8).

30 See Dugald Baird, ‘A Fifth Freedom?’ (1965) 5471 BMJ 1141, 1141; Gayle Davis and Roger Davidson, ‘“The Fifth Freedom” or “Hideous Atheistic Expediency”: The Medical Community and Abortion Law Reform in Scotland, c.1960–75’ (2006) 50 Medical History 29. See further David Steel, *Against Goliath: David Steel’s Story* (Pan Books 1991) 64.

31 Alice Jenkins, *Law for the Rich: A Plea for the Reform of the Abortion Law* (Victor Gollancz 1960); Steel (n 30) 60–1; HC Deb 13 July 1967, vol 750, col 1349.

32 HC Deb 13 July 1967, vol 750, col 1348.

33 See generally Keown (n 19); Sheldon (n 8); Sheelagh McGuinness and Michael Thomson, ‘Medicine and Abortion Law: Complicating the Reforming Profession’, (2015) 23 Med L Rev 177.

34 Dr Michael Winstanley, HC Deb 29 July 1967, vol 749, col 1059.

35 See Sheldon and others (n 8) Appendix 2.

36 See generally ibid; John Curtice and others (eds), *British Social Attitudes: The 36th Report* (Natcen Social Research); S Frankenburg and others*, British Social Attitudes: The 40th Report* (Natcen Social Research). See further Ben Clements and Clive Field, ‘Abortion and Public Opinion in Great Britain: A 50-year Retrospective’ (2018) 39 Journal of Beliefs & Values 429. On the declining influence of religion, see eg Callum G Brown, *The Death of Christian Britain: Understanding Secularisation 1800–2000* (2nd edn, Routledge 2009); Callum G Brown, *Religion and the Demographic Revolution: Women and Secularisation in Canada, Ireland, UK and USA since the 1960s* (Boydell Press 2012).

37 See generally Pam Lowe and Sarah-Jane Page, *Anti-Abortion Activism in the UK: Ultra-sacrificial Motherhood, Religion and Reproductive Rights in the Public Sphere* (Emerald 2022); Olivia Dee, *The Anti-Abortion Campaign in England, 1966–1989* (Routledge 2020); Drew Halfmann, *Doctors and Demonstrators: How Political Institutions Shape Abortion Law in the United States, Britain and Canada* (University of Chicago 2011); Sheldon and others (n 8).

38 See generally Lowe and Page (n 37); Sheldon and others (n 8).

39 For further discussion of both changes, see Sheldon and others (n 8).

40 RCOG, *The Care of Women Requesting Induced Abortion* (RCOG Evidence-Based Clinical Guideline No 7, 2011); NICE, ‘Abortion Care’ (NICE Guideline [NG140], 2019) www.nice.org.uk/guidance/ng140.

41 See Department of Health and Social Care, *Abortion Statistics for England and Wales: 2020* (2022).

42 The proportion who agree with this statement grew from 37% in 1983 (with over half believing it should not) to 76% in 2023, see Elizabeth Clery, ‘A Liberalisation in Attitudes’ (British Social Attitudes 40, The National Centre for Social Research 2023). See further Clements and Field (n 36).

43 Nicole Stone and Roger Ingham, ‘Who Presents More than Once? Repeat Abortion among Women in Britain’ (2011) 37 Journal of Family Planning and Reproductive Health Care 209.

44 Sheldon and others (n 8) ch 3.

45 Cornerstone Group, ‘About Us’ <https://cornerstonegroup.wordpress.com/about/> accessed 23 May 2023.

46 Sheldon and others (n 8) ch 6.

47 See Ilana Löwy, ‘Prenatal Diagnosis: The Irresistible Rise of the “Visible Foetus”’ (2014) 47(B) Studies in History of Philosophy of Biological and Biomedical Sciences 290, 290; Ilana Löwy, ‘Prenatal Diagnosis, Surveillance and Risk’ in Nick Hopwood, Rebecca Flemming and Lauren Kassell (eds), *Reproduction: Antiquity to the Present Day* (CUP 2018) 567, 568; Wright (n 4).

48 From an estimated risk of 3–5% in the early 1970s to less than 0.5% today. See Lane, *Report of the Committee on the Working of the Abortion Act* (Cmnd 5579, 1974) Appendix to s J, 258; RCOG, *Amniocentesis and Chorionic Villus Sampling* (RCOG Green-Top Guideline No 8, updated 2021). See generally contributions to EM Tansey and DA Christie (eds), *Looking at the Unborn: Historical Aspects of Obstetric Ultrasound* (Wellcome Witnesses to Twentieth Century Medicine Seminar Transcript, 2000); MB McNay and JE Fleming, ‘Forty Years of Obstetric Ultrasound 1957–97’ (1999) 25 Ultrasound in Medicine and Biology 3.

49 NIPT tests fetal DNA in maternal blood and is offered where screening indicates an increased possibility of Down’s, Edwards’s or Patau’s syndromes. For a useful chronology and more explanation of these developments, see Nuffield Council on Bioethics, *Non-Invasive Prenatal Testing: Ethical Issues* (2017). See further NJ Wald and others, ‘Maternal Serum Screening for Down’s Syndrome in Early Pregnancy’ (1988) 297 BMJ 883; Attie TJI Go, John MG van Vugt and Cees BM Oudejans, ‘Non-Invasive Aneuploidy Detection Using Free Fetal DNA and RNA in Maternal Plasma: Recent Progress and Future Possibilities’ (2011) 17 Human Reproduction Update 372; ‘NHS to Offer Safer Down’s Syndrome Test to Pregnant Women’ *The Guardian* (London, 29 October 2016).

50 See generally Thomas (n 25).

51 In 2018, 44% of women offered screening declined it: *Crowter v SSHSC* EWCA Civ [2022] 1559, [20].

52 Rates varied from 89% to 95% between 1989 and 2012, see JK Morris and A Springett, *The National Down Syndrome Cytogenetic Register for England and Wales: 2013* (National Down Syndrome Cytogenetic Register 2014).

53 St John-Stevas (n 27).

54 C Richards, *A World Without Down Syndrome?* (BBC2, 8 November 2016); General Synod of the Church of England, ‘Valuing People with Down’s Syndrome’ (9 January 2018). See generally Löwy, ‘Prenatal Diagnosis: The Irresistible Rise of the “Visible Foetus”’ (n 47) 290; Thomas (n 25) 77. PADS, *Sharing the News: The Maternity Experience of Parents of a Baby with Down Syndrome* (2019) <https://righttolife.org.uk/wp-content/uploads/2021/02/PADS-brochure-for-screen.pdf> accessed 15 November 2023.

55 See generally Anne Borsay, *Disability and Social Policy in Britain Since 1750* (Palgrave 2005); Committee on the Rights of Persons with Disabilities, *Inquiry concerning the United Kingdom of Great Britain and Northern Ireland carried out by the Committee under Article 6 of the Optional Protocol to the Convention. Report of the Committee* (2016) CRPD/C/15/R.2/Rev.1; Laura Abreu, *The UN Convention on the Rights of Persons with Disabilities: UK Implementation* (House of Commons Library Research Briefing, 10 November 2022). On recent setbacks, see Frances Ryan, *Crippled: Austerity and the Demonization of Disabled People* (Verso 2020).

56 Archives of the Royal College of Obstetricians and Gynaecologists, ‘New Test Could Halve Down’s Syndrome Births’ (Press Release RCOG/N4/1993, July 1993). On the pervasiveness of such assumptions in the early history of prenatal testing, see Löwy, ‘Prenatal Diagnosis: The Irresistible Rise of the “Visible Foetus”’ (n 47) 290–9.

57 eg RCOG, *Amniocentesis and Chorionic Villus Sampling* (n 48); RCOG and others, *Supporting Women and Their Partners through Prenatal Screening for Down’s Syndrome, Edwards’ Syndrome and Patau’s Syndrome. Consensus Statement from the Royal College of Obstetricians and Gynaecologists, Royal College of Midwives, Society and College of Radiographers* (December 2020); RCOG, *Termination of Pregnancy for Fetal Abnormality in England, Scotland and Wales* (2010); NHS, *Fetal Anomaly Screening Programme Handbook* (2015, updated 2022). See also Public Health England, *Fetal Anomaly Screening Programme—Screening for Down’s syndrome, Edwards’ syndrome and Patau’s syndrome (Trisomy 21, 18 & 13)* (NHS Service Specification No 16, 2019–20).

58 Sheldon (n 19).

59 Sheldon and others (n 8) 139–43, 170–6, 241–4. In 2008, measures seeking to prohibit abortion for reason of disability, gender, race or sexual orientation of the future child or for fetal anomaly were tabled by the Catholic Vice-Chairs of the APPG. A subsequent APPG Chair tabled a Presentation Bill that proposed amending the Abortion Act explicitly to exclude cleft lip, cleft palate and clubfoot as grounds for abortion. See Abortion (Cleft Lip, Cleft Palate and Clubfoot) Bill (2020), HC Deb 3 June 2020, vol 676, col 887.

60 Fiona Bruce, ‘Parliamentary Inquiry into Abortion on the Grounds of Disability’ (2013) <http://dontscreenusout.org/wp-content/uploads/2016/02/Abortion-and-Disability-Report-17-7-13.pdf> accessed 15 November 2023.

61 Archbishop Cranmer, ‘Kevin (Lord) Shinkwin Appointed to Equality and Human Rights Commission’ (2017, blog no longer available).

62 Abortion (Disability Equality) Bill [HL] 2016/17, ‘Tory Peer Lord Shinkwin Warns Britain’s Abortion Laws Are a “Licence to Kill Disabled People”, *The Telegraph* (London, 10 March 2017).

63 Abortion (Disability Equality) Bill [HL] 2017, also proposing that ‘full and accurate information’ should accompany any positive prenatal diagnosis of a fetal anomaly, to include ‘material written by groups representing people with experience of the anomaly in question’.

64 Witness Statement of Lord Shinkwin in Core Bundle (n 5).

65 Celia Hall, ‘Disabled Group in Abortion Law Attack’ *The Telegraph* (London, 22 August 2001). The Disability Rights Commission was founded in 2000 and replaced by the Equality and Human Rights Commission in 2007.

66 Interview in Gaby Hinsliff, ‘‘I Want to Wake up this Nation’s Conscience’ *The Observer* (London, 27 February 2005).

67 *R (Jepson) v Chief Constable of the West Mercia Police Constabulary* [2003] EWHC 3318. BBC News, ‘Curate Wins Abortion Challenge’ (1 December 2003); Barbara Hewson, ‘Clinical Negligence: Denied Access’ *Legal Week* (7 December 2005).

68 BBC News (n 67); BBC News, ‘Police Examine “Cleft Palate” Abortion’ (28 October 2002).

69 Hewson (n 67); BBC News, ‘No Charges in Late Abortion Case’ (16 March 2005).

70 *In the Matter of an Appeal to the Information Tribunal under Section 57 of the Freedom of Information Act* 2000 No EA 2008/0074, [19], [62] (*FOIA*). See further ‘Doctor May be Charged over Late Abortion’ *Daily Mail* (London, 23 September 2004).

71 Jane Fisher, ‘Post-24 Week Termination for Fetal Anomaly’ in BPAS, *Britain’s Abortion Law* (2013) 29; *FOIA* (n 70); see further Sheldon and others (n 8) 174.

72 Judicial Review Claim Form; Statement of Facts and Grounds (n 14) [2], [70]–[71].

73 See nn 62–3.

74 Statement of Facts and Grounds (n 14) [3].

75 ibid [50]–[56].

76 ibid [31], [48].

77 ibid [64]–[66].

78 ibid [67].

79 *Crowter v SSHC* [2021] EWHC 2536, [62].

80 ibid [62]–[71] on art 2, [72]–[83] on art 3.

81 Statement of Facts and Grounds (n 14) [28].

82 ibid [21], [28].

83 Witness Statement of Professor Katrina Scior, Professor Clinical Psychology and Stigma Studies, UCL, in Core Bundle (n 5).

84 *Aksu v Turkey* (2013) 56 EHRR 4, [58].

85 Second Witness Statement of Máire Lea-Wilson in Core Bundle (n 5) [13], [15], citing from articles about the storyline published in *The Times* and *Daily Mail* and on Mumsnet and Twitter, and reporting the findings of a small online survey.

86 ibid [29].

87 Witness Statement of Máire Lea-Wilson, in Core Bundle (n 5) [14].

88 ibid [15].

89 ibid [17].

90 ibid [18].

91 ibid [21].

92 ibid [22].

93 ibid [23].

94 Statement of Facts and Grounds (n 14) [30]; PADS (n 52).

95 Statement of Facts and Grounds (n 14) [19]–[23].

96 Witness Statement of Máire Lea-Wilson (n 87) [2], [26]–[27].

97 *Crowter* (n 77) [102], including the Equality Act 2010.

98 ibid [100]–[103].

99 ibid [126].

100 ibid [84]–[135] on art 8, [136]–[147] on art 14. In particular, the Court had heard no evidence from ‘women whose choices would be curtailed (and potentially made criminal)’ by any change in the law, ibid [130].

101 ibid [9].

102 ibid [150].

103 This would neither have affected the validity of the Abortion Act nor been binding on the parties, ibid [151]–[155].

104 ibid [31].

105 Appellants’ Replacement Skeleton Argument (7 June 2022) on file with the author, citing *Aksu v Turkey* (2013) 56 EHRR 4, [58].

106 ibid [32]–[33], citing *R (Bridges) v Chief Constable of South Wales Police* [2020] 1 WLR 5037, [55] and noting that the problem was not solved by any professional guidance, which was ‘explanatory rather than prescriptive’.

107 ibid.

108 *Crowter* (n 51) [70].

109 ibid (Underhill LJ) [57]–[58], although Peter Jackson LJ accepted that ‘institutional stigma’—such as that inherent in legislation—can play a powerful role in countering or promoting negative stereotypes, prejudice and discrimination, ibid [36].

110 ibid [57].

111 ibid (Peter Jackson LJ) [129], taking account also of the need for a balance to be struck between the rights of pregnant women and the interests of the unborn, that the current balance has been struck by democratic means and that the legislation does not directly concern the appellants, is not intended to cause offence and is not the root cause of discrimination but one of many contributors to it.

112 ibid (Underhill LJ) [73], (Peter Jackson LJ) [129].

113 ibid (Peter Jackson LJ) [129].

114 ibid (Thirlwall LJ) [98].

115 ibid [127].

116 Matthew Weaver, ‘Woman with Down’s Syndrome Loses Court of Appeal Abortion Law Case’ *The Guardian* (London, 25 November 2022).

117 Don’t Screen Us Out, ‘Woman with Down’s Syndrome to Take Case against UK Govt over Discriminatory Abortion Law to European Court of Human Rights’ (Press Release 18 May 2023) <https://dontscreenusout.org/press-release-woman-with-downs-syndrome-to-take-case-against-uk-govt-over-discriminatory-abortion-law-to-european-court-of-human-rights> accessed 27 May 2023.

118 Sheldon and others (n 8) ch 5.

119 *Paton v BPAS* [1978] 2 All ER 987, 989. Compare the *obiter* comments and findings of Lord Denning MR in *Royal College of Nursing v Department of Health and Social Security* [1981] AC 800, Cooke J in *R v Catt* (unreported, 17 September 2012) and Munby J in *R (Smeaton) v Secretary of State for Health* [2002] EWHC 610. On these cases generally, see Sheldon and others (n 8); on how judges’ understanding of public policy may influence their decisions, see eg Richard A Posner, ‘Legal Formalism, Legal Realism, and the Interpretation of Statutes and the Constitution’ (1986) 37 Case W Res L Rev 179.

120 For example, Gerard Wright QC published an argument for a more restrictive interpretation of abortion law long before securing the opportunity to test it in the courts in support of Robert Carver’s attempt to prevent his ex-girlfriend from terminating her pregnancy in *C v S* [1987] 1 All ER 1230, see Gerard Wright (1981) ‘Capable of Being Born Alive?’ 131(5989) NLJ 188. Wright acted in a number of other important cases of interest to Christian groups, including *R v Salford Health Authority ex parte Janaway* [1988] 3 All ER 3 1079 and *Gillick v Wisbech AHA* [1986] AC 112. Wright described himself as an ‘unconditionally Pro-Life’ lawyer, Gerard Wright ‘The Culture of Death’ [1999] Catholic Medical Quarterly 19.

121 *R (Axon) v Secretary of State for Health* [2006] EWHC 37; *R (A and B) v Secretary of State for Health* [2017] UKSC 41; *Re NIHR* [2018] UKSC 27.

122 On *Jepson*, see nn 66–71 and accompanying text. The ProLife Alliance is an anti-abortion single-issue political party that fielded parliamentary candidates in 1997 and 2001. Quintavalle was responsible for co-drafting the initial grounds of claim and grounds of appeal and assisting in the Court of Appeal, but was replaced for the High Court hearing.

123 Quintavalle brought an action against the Human Fertilisation and Embryology Authority in his own name (*R v Secretary of State for Health ex parte Quintavalle (on behalf of Pro-Life Alliance)* [2003] 2 WLR 692) and has acted professionally in a number of cases regarding withdrawal of life support from severely impaired children, which have become an important focus for some Catholic and Evangelical Christian groups, see *Dance v Barts Health NHS Trust & Anor (Re Archie Battersbee)* [2022] EWCA Civ 1106; *Evans v Alder Hey Children’s NHS Foundation Trust* [2018] EWCA Civ 984. Conrathe acted for the Christian Institute in threatening the BBC with a judicial review of its decision to air content ‘deeply offensive to Christians’ in *Jerry Springer—The Opera* (see ‘BBC Faces Legal Threat over Springer Broadcast’ *The Guardian* (London, 25 January 2005); for Lesley Burke in *R (Burke) v GMC* [2004] EWHC 1879 (Admin); and for Keira Bell in *Bell v Tavistock and Portman NHS Foundation Trust* [2020] EWHC 3274. He worked with the ProLife Alliance on litigation regarding the homosexual age of consent, see Paula Rohan, ‘Lawyer in the News’ *Law Society Gazette* (9 April 2001); and represented Nottingham Students for Life in their demand to be affiliated to the Student’s Union, Cathal McNaughton, ‘Anti-Abortion Student Group Hails Free Speech Victory’ *The Times* (London, 24 July 2019).

124 *R (Axon)* (n 121); Manchester Evening News staff, ‘Taxpayers Foot Bill for Abortion Case’ *Manchester Evening News* (Manchester, 24 January 2006).

125 Rebecca Allison, ‘Search for Woman after Court Rules on Abortion Attempt’ *The Guardian* (London, 21 March 2001).

126 Stephen Adams, ‘Why Wasn’t I Told the Baby I Aborted at 23 Weeks Might Be Able to Feel Pain?’ *Mail on Sunday* (London, 3 May 2020).

127 Stuart Scheingold and Austin Sarat, *Something to Believe In: Politics, Professionalism, and Cause Lawyering* (Stanford UP 2004); Austin Sarat and Stuart Scheingold, *Cause Lawyering: Political Commitments and Professional Responsibilities* (OUP 1998); Andrew Boon, ‘Cause Lawyers and the Alternative Ethical Paradigm: Ideology and Transgression’ (2004) 7 *Legal Ethics* 250.

128 ‘Treasury Devil’ is the First Junior Treasury Counsel (Common Law), a private practitioner who represents the Government in the civil courts. The juniors were Julia Smyth and Yaaser Vanderman.

129 Detailed Grounds of Defence, in Core Bundle (n 5).

130 See nn 58–63 and accompanying text.

131 Statement of Facts and Grounds (n 14) [58].

132 ibid [49], [56].

133 [2001] 2 WLR 480, cited in ibid [61].

134 The three Court of Appeal judges who heard *Re A* offered different grounds for finding that separation surgery would be lawful. However, Walker LJ (ibid) emphasised ‘the unique circumstances for which this case is authority’: ‘it must be impossible to preserve the life of X. [the stronger conjoined twin or by analogy the pregnant woman] without bringing about the death of Y. [the weaker twin or, by analogy, fetus], that Y. by his or her very continued existence will inevitably bring about the death of X. within a short period of time, and that X. is capable of living an independent life but Y. is incapable under any circumstances (including all forms of medical intervention) of viable independent existence.’

135 eg National Advocates for Pregnant Women, ‘When Fetuses Gain Personhood: Understanding the Impact on IVF, Contraception, Medical Treatment, Criminal Law, Child Support, and Beyond’ (17 August 2022); Lynn M Paltrow and Jeanne Flavin, ‘Arrests of and Forced Interventions on Pregnant Women in the United States, 1973–2005: Implications for Women’s Legal Status and Public Health’ (2013) 38 Journal of Health Politics, Policy and Law 299.

136 RCOG, *Fetal Awareness: Report of a Working Party* (1997); RCOG, *Fetal Awareness—Review of Research and Recommendations for Practice* (2010); All-Party Parliamentary Pro-Life Group, *Foetal Sentience and Pain: an Evidence Review* (2020) <https://lordalton.files.wordpress.com/2020/03/2020-pro-life-appg-report-on-foetal-pain.pdf> accessed 30  December 2022.

137 Witness Statement of Professor John Wyatt in Core Bundle (n 5); Statement of Facts and Grounds (n 14) [64]–[66].

138 See British Medical Association, *The Law and Ethics of Abortion***—***BMA Views* (2020) 8.

139 Subject possibly to the very narrowly drawn exception in *Re A* (n 133).

140 Witness Statement of Nicola Enoch, in Core Bundle (n 5) [34]. The Ups of Downs offers services to families in Warwickshire; PADS provides information to complement the ‘over-medicalised information’ available in maternity units, ibid [2], [9].

141 Recommendations include the need for cultural change to address systemic discrimination in maternity services; results to be delivered in a non-directive, non-judgmental and supportive way, with no assumption that women will choose termination and all decisions respected; rapid access to appropriate counselling; signposting to local and national support organisations, and local families; staff training on the reality of living with Down’s syndrome in modern Britain; and balanced counselling. However, the report’s recommendations do differ from RCOG guidance in suggesting that the option of abortion should not be volunteered, with counselling not to focus on ‘what options are available, unless this information is requested’. dsuk, ‘PADS: Sharing the News: The Maternity Experience of a Parents of a Baby with Down Syndrome’ (2019) 9 < https://downsyndromeuk.co.uk/docs/Maternity/12197%20Sharing%20the%20news.pdf> accessed 27 October 2023.

142 Recommendations include that women or couples considering NIPT be offered opportunities to understand more about the lives of individuals with Down’s syndrome (eg through meetings); that further research be carried out into how best to support fully informed choice; and that people with Down’s syndrome be included in discussion in this area. Barbara Barter and Richard Hastings, *Consultation with Individuals with Down syndrome about Non Invasive Prenatal Testing (NIPT)* (MENCAP 2017).

143 ibid.

144 eg Melanie Symonds, *And Still They Weep* (SPUC Educational Research Trust 1996). See generally Reva B Siegel, ‘The Right’s Reasons: Constitutional Conflict and the Spread of Woman-Protective Anti-Abortion Argument’ (2008) 57 Duke LJ 1641; on the ‘women protective’ framing of modern anti-abortion campaigning, see Sheldon and others (n 8).

145 Jenkins (n 31).

146 Witness Statement of Professor Richard Hastings, in Core Bundle (n 5).

147 Committee on the Rights of Persons with Disabilities, *Report on the UK* (2017) CRPD/C/GBR/CO/1.

148 Above n 66.

149 See the Divisional Court’s summary of the evidence, *Crowter* (n 79) [9].

150 See Witness Statement of Andrea Duncan, then Head of Policy for Alcohol, Sexual and Reproductive Health, Department of Health and Social Care, in Core Bundle (n 5) [42]: one abortion was reported on the basis of Down’s syndrome in conjunction with other conditions at each of 21, 29 and 33 weeks, and one for Down’s syndrome alone at each of 30 and 32 weeks.

151 See Department of Health and Social Care, *Abortion Statistics for England and Wales: 2020* (2022) Table 9a, showing far larger numbers performed for malformations of the cardiovascular system (*n* = 53) or brain defects (five abortions after 24 weeks reported following diagnoses for each of hydrocephaly, microcephaly and anencephaly and 89 for other defects of the brain) and 43 for malformations of the musculoskeletal system. More than one condition was notified in some of these cases.

152 Witness Statement of Basky Thilaganathan, Director of Fetal Medicine at St George’s Hospital NHS Foundation Trust, and RCOG Council Member, in Core Bundle (n 5) [71].

153 Abortion Act 1967, s 1(1)(a).

154 John Pring, ‘Mixed Response from Disabled Activists to Heidi Crowter Abortion Case Ruling’ *DNS* (30 September 2021), citing, *inter alia*, the Greater Manchester Coalition of Disabled People, who praised Crowter’s ‘powerful activism’ but argued that ‘challenging abortion law is not the best way forward, as this could undermine rights’ and that ‘[g]enetic screening, and how things proceed following diagnosis, are the key discriminations here’.

155 Statement of Facts and Grounds (n 14) [37].

156 Joint Statement by the UN Committee on the Rights of Persons with Disabilities and the Committee on the Elimination of All Forms of Discrimination against Women, 29 August 2018, ‘Guaranteeing Sexual and Reproductive Health and Rights for All Women, in Particular Women with Disabilities’.

157 ibid.

158 Elizabeth Tilley and others, ‘“The Silence is Roaring”: Sterilization, Reproductive Rights and Women with Intellectual Disabilities’ (2012) 27 Disability & Society 413; Virginia Kallianes and Phyllis Rubenfeld, ‘Disabled Women and Reproductive Rights’ (1997) 12 Disability & Society 203.

159 See generally Elizabeth Dietz, ‘Abortion, Disability Rights and Reproductive Justice’ in Joel Michael Reynolds and Christine Wieseler (eds), *The Disability Bioethics Reader* (Routledge 2022); Marsha Saxton, ‘Disability Rights and Selective Abortion’ in Lennard J Davis (ed), *The Disability Studies Reader* (Taylor & Francis 2013).

160 For a discussion of how prenatal testing rendering societal-scale outcomes relates to individual choice, see Dietz (n 159) 96–7. See further Jackie Leach Scully, ‘Being Disabled and Contemplating Disabled Children’ in Reynolds and Wieseler (n 159) 116.

161 See further Bonnie Steinbock, ‘Disability, Prenatal Testing and Selective Abortion’ in Erik Parens and Adrienne Asch (eds), *Prenatal Testing and Disability Rights* (Georgetown UP 2000); Dietz (n 159); Saxton (n 159).

162 Thomas (n 25) esp ch 4.

163 On the complexity of decision making and limitations of existing diagnostic tools, see ibid; Witness Statement of Basky Thilaganathan (n 152) [72]–[77]; on the ‘sin of synecdoche’, where one part (disability) is made to stand in for the whole (a person), see Adrienne Asch and David Wasserman, ‘Where Is the Sin in Synecdoche? Prenatal Testing and the Parent–Child Relationship’ in David Wasserman, Jerome Bickenbach and Robert Wachbroit (eds), *Quality of Life and Human Difference* (CUP 2005).

164 Lea-Wilson complained that the obstetrician had written in her notes ‘parents are committed to the pregnancy’, which she felt dehumanised her baby (n 87) [19].

165 See generally Thomas (n 25).

166 Tom Shakespeare, ‘Foreword’ in Nuffield Council on Bioethics, *Non-Invasive Prenatal Testing: Ethical Issues* (2017).

167 World Health Organization, *Abortion Care Guideline* (2022) esp Recommendation 2.1.

168 For further discussion of the form that decriminalisation might take, see generally Sally Sheldon and Kaye Wellings (eds), *Decriminalising Abortion in the UK: What Would It Mean?* (Policy Shorts, Bristol UP 2020).

169 Criticism of the statutory language saw MENCAP file a witness statement as the case reached the Court of Appeal, focusing on the terminology used in s 1(1)(d) and attacking the provision as ‘an offensive anachronistic anomaly in the legislative landscape’, *Crowter* (n 51) [54].

170 See eg Women With Disabilities Australia, *Submission on Sexual and Reproductive Rights of Women and Girls with Disability to the Royal Commission into Violence, Abuse, Neglect and Exploitation of People with Disability* (December 2022).

171 See Brooke Ronald Johnson Jr, Louise Keogh and Wendy Norman, ‘What Would Be the Likely Impact of Decriminalisation on the Incidence, Timing, Provision and Safety of Abortion?’ in Sheldon and Wellings (n 168).

172 The authoritative British Social Attitudes survey does not ask a question framed in terms of fetal personhood. However, it seems reasonable to assume that the 95% of British adults who believe that abortion should be permitted where a woman’s health is seriously endangered and, *a fortiori*, the 76% who believe it should be permitted where a woman ‘decides on her own she does not wish to have a child’ do not believe a fetus to be a person with rights equivalent to those of the woman, see Clery (n 42).

173 Weaver (n 116).

174 Cited in CNA staff (n 6).

